# Impact of climate change on Boro rice production in Bangladesh: Evidence from time series modeling

**DOI:** 10.1371/journal.pone.0328699

**Published:** 2025-07-23

**Authors:** Rafee Shahrier, Mohammad Nazmol Hasan, Sadia Yesmin Ankita, Ismat Tasnim, Kazi Tamim Rahman

**Affiliations:** 1 Faculty of Agricultural Economics and Rural Development, Gazipur Agricultural University, Gazipur, Bangladesh; 2 Department of Agricultural and Applied Statistics, Bangladesh Agricultural University, Mymensingh, Bangladesh; 3 Department of Agricultural and Applied Statistics, Gazipur Agricultural University, Gazipur, Bangladesh; 4 Department of Agricultural Statistics, Sher-e- Bangla Agricultural University, Dhaka, Bangladesh; 5 Department of Agribusiness and Marketing, Bangladesh Agricultural University, Mymensingh, Bangladesh; 6 Department of Agricultural Economics, Gazipur Agricultural University, Gazipur, Bangladesh; Chandigarh University UCRD: Chandigarh University University Centre for Research & Development, INDIA

## Abstract

Bangladesh has three distinct rice-growing seasons: Aus, Aman, and Boro, each with its distinct climatic state. Climatic factors interacting with non-climatic factors impact seasonal rice yield. However, research hasn’t yet examined how climatic and non-climatic factors (CNCFs) affect the yield of rice production during the Boro season (YBR). Therefore, this study attempted to assess the impact of CNCFs on YBR using time series modeling. Accordingly, the modeling approaches used stationarity testing and pairwise correlation analysis to verify the suitability of the CNCFs for further analysis. After that, the autoregressive distributed lag (ARDL) model, the Granger causality test, and the principal component analysis (PCA) were used to predict how the CNCFs affect YBR. The ARDL model predicted that area and temperature had a substantial positive effect on YBR in both the long- and short-run, but humidity adversely influenced YBR in the long-run and positively in the short-run. The Granger causality test revealed a unidirectional causal relationship between YBR and CNCFs, except for the climatic factor rainfall. On the other hand, the non-climatic factors area, population, energy consumption, and fertilizer consumption were positively associated with YBR and substantially contributed to PC1’s (71.7%) variation. Aligning these results, this study concluded that the area, temperature, population, fertilizer consumption, and energy consumption positively impacted the YBR, while humidity negatively impacted it. These findings are crucial for ensuring Bangladesh’s rice security amid climate change, guiding policymaking, and addressing future rice demand. Therefore, policymakers and stakeholders should focus on controlling greenhouse gas emissions to keep temperatures and humidity consistent, developing climate-tolerant rice cultivars, encouraging farmers to use organic fertilizer, and adapting eco-friendly technologies for sustainable rice production.

## 1. Introduction

Rice is a staple food for almost 4 billion people globally, covering 27% of total calories in low- and middle-income nations. The world’s rice consumption is expected to rise from 479 million metric tons of milled rice in 2014–536–551 million metric tons in 2030, and to 584 million metric tons in 2050 [1,2]. Similarly, in Bangladesh, rice accounts for more than half of the average person’s protein intake, two-thirds of total calories, and over 48% of jobs in rural areas. The rice industry contributes one-sixth of Bangladesh’s overall national income and half of its agricultural GDP. Rice occupies approximately 75% of the farmed land and over 80% of the irrigated area [3,4]. In addition, rice is a rich source of more than fifteen essential vitamins and minerals, and its whole grains, such as brown rice, have the potential to prevent heart disease, diabetes, and specific forms of cancer [5,6].

Regardless of the importance of rice, its production is under threat due to climate change (CC). Higher temperatures, increased precipitation, and frequent extreme weather events due to CC impact on agricultural production, especially rice production. These factors intensify abiotic stresses like drought and salinity and reduce the number of beneficial microorganisms in the soil. Additionally, they exacerbate biotic stressors, such as insect and disease infestations, which are the cause of optimal plant growth and development. This results drastic drop in around half of the entire production of rice and other crops [6–19]. Bangladesh, being a low-lying country, is susceptible to flooding and sea level rise, making it extremely vulnerable to the impacts of CC. This is because of the Himalaya’s largest mountain range in the north, and the funnel-shaped Bay of Bengal in the south of Bangladesh [20,21]. Thus, the nation experiences high temperatures, heavy rainfall, and seasonal variations due to its geographical location. According to UNDP reports and BMD, the nation has experienced increased temperatures over the past thirty years, especially during the monsoon season [22–24]. In addition, an increase in annual mean temperatures of 1 °C is predicted for the nation by 2030, 1.4 °C by 2050, and 2.4 °C by 2100 [25]. Nonetheless, the forecast for the average temperature throughout the winter also indicated a similar upward trend, with 1.1 °C by 2030, 1.6 °C by 2050, and 2.7 °C by 2100. However, for the monsoon season, the estimated values are 0.8 °C by 2030, 1.1 °C by 2050, and 1.9 °C by 2100 [26,27]. On the other hand, the growing human population (POP), a non-climatic factor, puts pressure on agriculture to increase food productivity. Consequently, conventional farming techniques prove inadequate, prompting a transition towards fertilizer consumption (FC), irrigation, and modern farming technology based on the Internet of Things [28–31]. As a result, FC and energy consumption (EC) in agriculture are important factors for improving rice productivity. In Bangladesh, rice production uses over 75% of fertilizer consumption [32], significantly increasing rice yield [33]. Furthermore, studies have shown that modern agricultural techniques that use more energy can improve rice production [34–36]. Consequently, CNCFs are changing as time progresses. However, various crop cultivation, including seasonal rice, requires unique climatic conditions and optimal utilization of non-climatic factors to maximize yield. Therefore, the CNCFs may impact YBR in Bangladesh.

Numerous studies have been conducted worldwide to assess the impact of CC or CNCFs on various crops, including rice [15,16,37–45]. These studies enriched the literature significantly, providing valuable insights for policymakers and stakeholders, since sustainable crop production relies on information-based, data-driven policymaking and active awareness among multiple stakeholders. However, the impact of CNCFs on rice production (RP) and YBR in Bangladesh was found to vary depending on the study length, location, econometric method used, and choice of CNCFs/variables [37,41,44,46,47]. Two main limitations incurred from reviewing the literature in assessing the impact of CNCFs on RP or YBR: one is the annual measurement of CNCFs, and another is the methodological issues. Because of climatic variation over the year, measurement of CNCFs and crop yield/production over their growing season may produce more trustworthy results than annual measurement. On the other hand, some of the CNCFs that are supposed to have an impact on RP or YBR cannot be included in the model due to methodological constraints. To overcome these limitations, this paper employed time series modeling combined with principal component analysis (PCA), assessing the impact of CNCFs on the yield of seasonal Boro rice (YBR) in Bangladesh, which has not been investigated yet.

## 2. Review of literature and research gap

### 2.1. Review of literature

Researchers worldwide are looking for the key CNCFs affecting agricultural productivity to develop policies promoting sustainable crop production and ensuring food security. Therefore, the authors of this study employed Google Scholar and ScienceDirect search engines to review scientific literature pertinent to the study’s objective. The authors divided the studies into two groups: the Bangladesh and Asian countries perspective, and the perspective on the non-Asian countries. Afterward, the pertinent literature was summarized according to the study period, location, main objective, the method used, and the key findings in Table 1 to identify the research/knowledge gap and frame the significance of this work in context.

**Table 1:** Impact of CNCFs on rice and cereal production in Bangladesh, Asia, and the rest of the world.

Author	Period	Study location	Main objective investigated	Methods	Key findings
**Bangladesh Perspective**					
Al Mamun et al. [41]	1970-2020	Bangladesh	Impact of CNCFs on BRP	Quantile Regression	***LI:*** MxT(+), MnT(+), AHBS(+), ABR(+).
Islam et al. [44]	1976-2015	Bangladesh	Impact of CC on BRP	Regression	***LI:*** AT(+), AH(-), AR(+), SS(-)
Iftekhar Uddin et al. [46]	1972-2014	Bangladesh	Impact of CC on BRP	Regression	***LI:*** MxT(-), MnT(+), AR(-)
Sarker et al. [47]	1972-2009	Bangladesh	Impact of CC on BRP	Regression	***LI:*** MxT(-), MnT(+)
Alam et al. [48]	1949-2013	Bangladesh	Impact of CC on RP	Regression	***LI:*** AR(-).
Sarker et al. [37]	1972-2009	Bangladesh	Impact of CC on RP	CDPF and Quadratic Function	***LI:*** MxT(+), MnT(-), AR(+)
Chandio et al. [39]	1988-2014	Bangladesh	Impact of CNCFs on CP	ARDL	***LI & SI:*** CO_2_(-), AT(-), AR(+), area(+), FD(+), EC(+).
Iqbal et al. [49]	1975-2008	Bangladesh	Impact of CC on CP	Regression	***LI:*** MnT Dry(+), MnT Wet(-), MxT Dry(-), MxT Wet(-).
Noorunnahar et al. [42]	1980-2020	Bangladesh	Impact of CNCFs on MP	ARDL	***LI:*** CO_2_(-), AT(-), AR(-), AgTech(+), POP(-), EC(-). ***SI:*** CO_2_(-), AT(-), AR(-), EC(+).
**Perspective on the Asian Countries**					
Li C. [43]	1978-2020	Japan	Impact of CC on RP	CDPF	***LI:*** AR(-), WS(-), SS(+).
Gul et al. [38]	1970-2018	Pakistan	Impact of CNCFs on RP	ARDL, FMOLS, CCR	***LI:*** AT(-), CO_2_(+), area(+), FC (+), LF(+), WA(+).
Pickson et al. [15]	1998-2017	China	Impact of CNCFs on RP	ARDL	***LI:*** AT(-), AR(+), area(+), FC(+). ***SI:*** AT(+), area(+).
Chandio et al. [40]	1961-2016	Asia	Impact of CNCFs on RP	DOLS	***LI:*** CO_2_(-), AT(-), AR(+), area(+), FC(+), L(+).
Abbas & Mayo [8]	1981-2017	Pakistan	Impact of CC on RP	CDPF	***LI:*** MxT(-), MnT(+), AR(-)
Guntukula R. [50]	1961-2017	India	Impact of CC on RP	Regression	***LI:*** AR(-), MxT(-), MnT(+)
Chandio et al. [51]	1990-2016	Nepal	Impact of CNCFs on RP	ARDL	***LI:*** CO_2_(-), AT(+), AR(+), area(+), FC(+), AC(+).
Chandio et al. [52]	1968-2014	Pakistan	Impact of CNCFs on RP	ARDL, FMOLS, CCR	***LI:*** CO_2_(+), area(+), FC(+). ***SI:*** CO_2_(+), AT(+), area(+).
Chandio et al. [13]	1965-2015	India	Impact of CNCFs on CP	ARDL	***LI:*** CO_2_(+), AR(+), AT(-), area(+), EC(+), FD(+). ***SI:*** CO_2_(-), AT(-), AR(+).
Ahsan et al. [53]	1971-2014	Pakistan	Impact of CNCFs on CP	ARDL	***LI:*** CO_2_(+), EC(+), area(+).
Chandio et al. [54]	1983-2016	Nepal	Impacts of CC on MP	ARDL	***LI:*** CO_2_(-), AT(-), AR(+). ***SI:*** CO_2_(-), AT(-), AR(+).
**Perspective on the Non-Asian Countries**					
Pickson et al. [16]	1970-2017	Africa	Impact of CC on CP	ARDL	***LI:*** AT(-), AR(+).
Sharma et al. [55]	1970-2020	Mississippi	Impact of CC on MP	ARDL	***LI:*** MxT(-), MnT(+), CO_2_(+).
Asfew & Bedemo [56]	1990-2020	Ethiopia	Impact of CC on CP	ARDL	***LI & SI:*** AR(+), AT(-), area(+), FC(+), CO_2_(+).
Maïga et al. [45]	1990-2020	Mali	Impact of CC on MP	ARDL	***LI & SI:*** AR(-), AT(-), area(+), GDP per capita(+).
Chandio et al. [57]	1968-2014	Turkey	Impact of CC on CP	ARDL	***LI & SI:*** CO_2_(-), AT(-), AR(+).

Note: (+), and (-) indicate positive and negative impacts respectively, long-run impact (*LI*), short-run impact (*SI*), rice production (RP), cereal production (CP), maize production (MP), average annual temperature (AT), average minimum temperature (MnT), average maximum temperature (MxT), average MnT in dry season (MnT Dry), average MnT in wet season (MnT Wet), average MxT in dry season (MxT Dry), average MxT in wet season (MxT Wet), average annual rainfall (AR), average annual humidity (AH), water availability (WA), sunshine (SS), wind speed (WS), carbon dioxide (CO_2_), fertilizer consumption (FC), labor force (LF), fertilizer consumption (FC), financial development (FD), agricultural credit (AC), Cobb-Douglas production function (CDPF), fully modified ordinary least squares (FMOLS), and canonical cointegration regression (CCR).

#### 2.1.1. Bangladesh and Asian perspective.

This section examined research done in Bangladesh and Asian contexts (Table 1) that shed light on how CNCFs affect RP, BRP, CP, and MP over various periods and econometric techniques. According to these research findings, national and international policies should be implemented to maintain favorable environmental conditions for sustainable agriculture.

In the context of Bangladesh, Al Mamun et al. [41], using quantile regression, discovered that MxT, MnT, AHBS, and ABR all had significant long-term beneficial impacts on BRP. However, Islam et al. [44] demonstrated by regression analysis that AH and SS had detrimental impacts on BRP, but AT and AR had beneficial impacts. Iftikhar Uddin et al. [46] and Sarker et al. [47], using regression analysis, forecasted that MnT had a favorable long-term effect on BRP, whereas MxT had a negative effect. Furthermore, Iftekhar Uddin et al. [46] discovered that AR had a favorable impact. According to regression results, AR has a detrimental impact on RP, as demonstrated by Alam et al. [48]. Sarker et al. [37] used regression to predict that MnT had a negative long-term influence on RP in Bangladesh, while MxT and AR were affected positively. Employing the ARDL approach, Chandio et al. [39] discovered that AR, area under production, FD, and EC had a favorable long-term and short-term impact on Bangladesh’s CP, while AT and CO2 had a negative influence. Iqbal et al. [49] also analyzed how, from 1975 to 2008, Bangladesh’s CP was negatively affected by the MnT Wet and the MxT Dry and MxT Wet and positively influenced by the MnT Dry. According to the ARDL model, Noorunnahar et al. [42] demonstrated that AgTech had a favorable impact on MP over the long term, while CO_2_, AT, AR, POP, and EC had a negative impact. However, only EC had a beneficial effect on MP in Bangladesh in the short term, while CO_2_, AT, and AR had a negative effect.

Likewise, different Asian countries had unique effects of CNCFs on RP patterns, and the research proposed several diverse strategic plans for sustainable production. As an example, CDPF Li C. [43] showed that SS had a long-term favorable effect on RP in Japan, but AR and WS had a negative effect. The influence of CNCFs on RP in Pakistan was summarized by Gul et al. [38] using the ARDL, FMOLS, and CCR models. In the long run, Pakistan’s RP was positively impacted by CO_2_, area, FC, LF, and WA, but AT had a negative impact. This suggests that national and international policies should balance the trade-offs between CO2 emissions, AT, and energy consumption to achieve sustainable RP. According to the ARDL model, Pickson et al. [15] predicted that while AT and area had a short-term positive influence on China’s RP, AT had a long-term negative impact, and AR, area, and FC had a positive impact. Chandio et al. [40], using DOLS, discovered that the RP for the Asia viewpoint was positively impacted by AR, area, FC, and LF, while CO₂ and AT had an undesirable effect. Following this, Abbas & Mayo [8] and Guntukula R. [50] investigated the long-term effects of MxT and AR negatively and MnT favorably on RP in Pakistan and India, respectively. Chandio et al. [51] and Chandio et al. [52] investigated the impact of CNCFs on Nepal and Pakistan’s RP in different periods using ARDL and ARDL, FMOLS, and CCR, respectively. In Pakistan, CO_2_ negatively and AT, AR, area, FC, and AC positively impacted RP in the long run. Nonetheless, Nepal’s RP was positively affected by CO_2_, area, and FC in the long run, while in the short run, CO_2_, AT, and area under cultivation also impacted positively. Chandio et al. [13] also investigated the impact of CNCFs on India’s CP in the long- and short-term. Their findings indicated that AT was the only factor with a negative impact, while CO₂, AR, area under cultivation, EC, and FD had positive impacts in the long term. However, in the short term, CO2 and AT were reported to have negative impacts, while AR was a positive factor. Similarly, Chandio et al. [54] used the ARDL model to forecast that the long- and short-term effects of CO₂, AT, and AR on Nepal’s MP would be the same as those in India. Furthermore, Ahsan et al. [53], using the ARDL technique, determined that CO₂, EC, and cultivated area had a positive impact on Nepal’s CP.

Finally, it is observed from the literature discussed here that, according to Bangladesh and Asian perspectives, there is a lack of agreement between studies regarding the impact of CNCFs on the BRP, RP, or CP. These disagreements arise due to the variation in the statistical methods used, choice of CNCFs, period considered, and locations that differ.

#### 2.1.2. Perspective on the non-Asian Countries.

Research examining the effects of CNCFs on the BRP, RP, or CP in non-Asian nations was evaluated in this section. Our search found studies that investigated the impact of CNCFs on the CP and MP. Unfortunately, no research was found that examined the effect of CNCFs on the BRP and RP. According to the ARDL model, Pickson et al. (2024) [16], Asfew & Bedemo (2022) [56], and Chandio et al. (2020) [57] discovered that, in the short and long terms, respectively, AR had a beneficial effect on the CP of Ethiopia, Africa, and Turkey, whereas AT had a negative effect. Additionally, Chandio et al. (2020) [57] discovered that CO_2_ had a negative effect on the CP of Ethiopia and Turkey, whereas Asfew & Bedemo (2022) [56] noted that the area under production, FC, and CO_2_ were had a positive effect. Sharma et al. (2023) [55] claimed that MxT has a long-term detrimental effect on Mississippi’s MP, while MnT and CO_2_ have a positive effect, based on data from 1970 to 2020 and the ARDL model. Using data from 1990 to 2020 and the ARDL model, Maïga et al. (2021) [45] calculated the short- and long-term effects of the CNCFs on Mali’s MP. They found that Mali’s MP was positively impacted by GDP and the area under MP, but negatively by AR and AT.

While some research studies agreed with their findings about the effects of certain CNCFs on CP in certain nations, some did not. Differences in the selection of CNCFs, the period taken into consideration, the choice of the variable, and the locations that differ lead to disparities.

### 2.2. Research gap

From the reviewed literature, it could be concluded that in Bangladesh, Asian, and non-Asian countries’ perspectives, the mode of the impact of CNCFs on BRP, RP, and CP was varied. This variation was even apparent in the same crop over identical regions. This variation is due to differences in the CNCF/variable selection, study period, location, and method used. Another source of this discrepancy is the measurement of time series variables. In certain instances, climatic factors were selected based on the crop-growing season, whereas in others, these factors were considered from an annual perspective. However, some crops, like rice, don’t need the entire year to grow from planting to harvesting. Accordingly, as a result of climatic fluctuations, there are three rice-growing seasons in Bangladesh over a year, namely Aus (March to August), Aman (June to December), and Boro (November to May) [58,59]. Consequently, in the Aus, Aman, and Boro seasons, the average temperature was 28.02^o^ C, 26.61^o^ C, and 23.91^o^ C [60,61]; the average rainfall was 310.45 mm, 276.19 mm, and 78.06 mm [62]; and the average humidity was 80%, 83%, and 76% [63], respectively. These examples demonstrate that the conditions of CNCFs vary over three rice-growing seasons, which may impact phenotypic characteristics, including the yield of these rice types in different patterns for Bangladesh. For example, in Bangladesh, in 2021–2022, the rice production shares during the Aus, Aman, and Boro seasons were 8%, 39%, and 53%, as well as cultivation areas occupied for these seasons were 10%, 49%, and 41%, respectively [64]. Furthermore, production contribution to the country’s Boro rice output increased by 0.97% annually between 1969–1970 and 2019–20, whereas the production contribution from the Aus and Aman seasons notably declined by 0.48% and 0.49% every year, respectively [65]. Different climatic states over the Aus, Aman, and Boro seasons may be one of the reasons for the yield performance of Aus, Aman, and Boro rice. On the other hand, the non-climatic factor growing human population, puts pressure on agriculture to increase food productivity, influencing the use of synthetic fertilizers and modern farming techniques that require more energy [28–36]. Therefore, the impact assessment of CNCFs on seasonal rice production will yield more accurate and trustworthy results than the impact of climate change on annual rice production. However, most of the studies in the literature evaluated the long- and short-run impact of CNCFs on gross annual rice or cereal production using the ARDL model and long-run impact using regression approaches [8,15,16,21,39,41,42,66–69]. On the other hand, a significant drawback of the regression approach is its failure to reflect the past behavior of time series data [70–72]. Furthermore, since the full model estimates of ARDL and regression sometimes become spurious if there is a strong correlation between the CNCFs [70–73], it’s better to use a subset of CNCFs in the ARDL or regression model. In this regard, PCA may be an important and useful alternative approach to evaluate the impact of the rest of the CNCFs on YBR, overcoming the limitations of multicollinearity when CNCFs are stationary at level I(0) or stationary at first difference I(1). Subsequently, regressing YBR on the important (principal component) PC scores, and the important CNCFs can be retrieved from the loadings or contribution of the CNCFs to the respective PC [71,74–79]. On the other side, the Granger causality analysis predicts the causal effects of one variable on another and vice versa, whether the variables are cointegrated or not [72,80–82]. This literature review identified two constraints in evaluating the impact of CNCFs on crop production, including YBR: one pertains to the annual measurement of CNCFs and crop yield, while the other arises from methodological considerations. Therefore, to overcome these limitations, this study suggests applying time series modeling, PCA, and Granger causality analysis to evaluate the impact of CNCFs on seasonal Boro rice yield (YBR) in Bangladesh, the nation’s most important seasonal rice in terms of area, production, and yield.

## 3. Materials and methods

**Fig 1:**
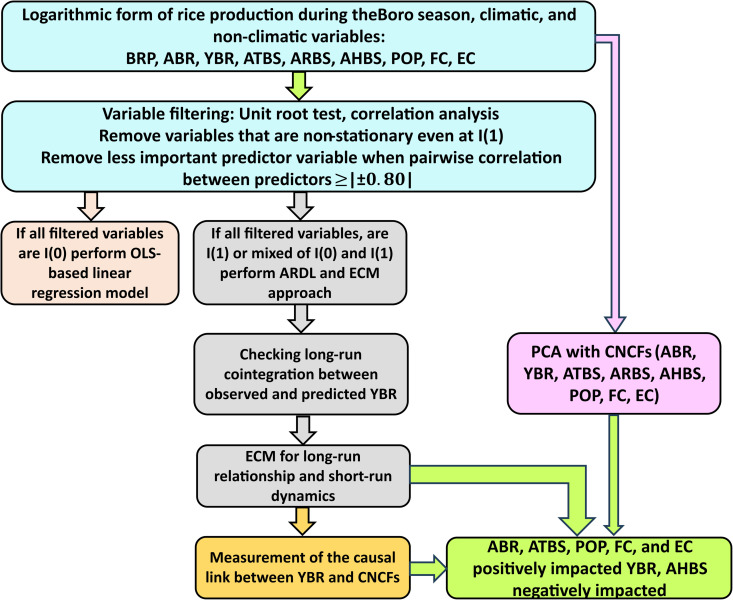
Workflow of time series modeling for impact assessment of CNCFs on YBR in Bangladesh.

This study provided statistical modeling guidelines for time series data to evaluate the impact of CNCFs on YBR in Bangladesh, acknowledging that a single approach was insufficient. After collecting and processing the time series data, the modeling pipeline primarily analyzed summary statistics, stationarity, and pairwise correlation. These analyses determined the candidate CNCFs included in the subsequent analysis, including ARDL, Granger causality, and PCA. Multiple R packages (supporting information: S1 Appendix), including ARDL [83] were used to analyze the data of this study. The study detailed the time series modeling guidelines in the following sections and Fig 1.

### 3.1. Data collection and processing

This study used time series data on CNCFs for Bangladesh from 1972 to 2019 (48 years), which includes data on rice production during the Boro season (BRP), area under BRP (ABR), YBR, and climatic factors average temperature during the Boro season (ATBS), average of the monthly total rainfall during the Boro season (ARBS), and average humidity during the Boro season (AHBS), as well as data on non-climatic factors or variables POP, FC, and EC. These data were provided by the Food and Agriculture Organisation (FAO), Bangladesh Meteorological Department (BMD), Bangladesh Rice Research Institute (BRRI), and Our World in Data (OWID) [84–88]. A detailed description of the data is given in Table 2. The YBR was computed by dividing the BRP by the ABR. We computed the average values of the meteorological variables, such as ATBS, ARBS, and AHBS, using raw data from 35 weather stations in Bangladesh.

**Table 2:** Variable description.

Variables	Acronym (log form of the original variable)	Measurement unit	Sources
Rice production during the Boro season	BRP	000’ton	BRRI
Area under BRP	ABR	000’ha	BRRI
Yield of BRP	YBR	ton/ha	BRRI
**Climatic variables**			
Average temperature during the Boro season	ATBS	Centigrade	BMD and BRRI
Average of the monthly total rainfall during the Boro season	ARBS	Millimeter (mm)	BMD and BRRI
Average humidity during the Boro season	AHBS	%	BMD and BRRI
**Non-climatic variables**			
Total population	POP	000 million	FAOSTAT
Fertilizer consumption	FC	Kg/ha	FAOSTAT
Energy consumption	EC	Terajoule	OWID

### 3.2. Descriptive statistics, correlation analysis, and stationarity checking

This study computed descriptive statistics, such as mean, median, and standard deviation (SD), minimum, maximum, first quartile (Q_1_), third quartile (Q_3_), and skewness to obtain the data structure. The relationship between the BRP, YBR, and CNCFs was obtained from the pairwise correlation analysis. Thereafter, Augmented Dickey-Fuller (ADF) and Phillips-Perron (PP) tests were used to find the unit root (non-stationarity) in the time series variables. Additionally, this study used the Zivot-Andrews Unit Root Test [89] and the Minimum Lagrange Multiplier Unit Root Test [90,91] for checking stationarity in the presence of single and multiple structural breakpoints (SB).

### 3.3. Time series modeling with the ARDL model

Time series data usually tends to be related to its previous values, and this phenomenon is termed the autoregressive (AR) feature [71,73]. On the other hand, a time series variable may also depend on other time series variables with their historical values, termed the distributed lag (DL) model [71–73,92]. Therefore, a time series variable may depend on both the AR and DL relationships, which can be expressed as the autoregressive distributed lag (ARDL) [70,72,92]. This study’s ARDL model takes the following form:


$$y_{t}=\beta_{0}+\sum_{i=1}^{k}\beta_{i,y}y_{t-i}+\sum_{j=1}^{m}\sum_{l=1}^{p_{j}}\beta_{j,l}x_{j,t-l}+e_{t}\cdots \;\;\cdots \;\;\cdots$$ (1)

In this equation $y_{t}$ is the dependent variable (YBR), $\beta_{0}$ is the constant or intercept of the model, $\sum_{i=1}^{k}{\beta_{i,y}y}_{t-i}$ is the AR component, $\sum_{j=1}^{m}\sum_{l=1}^{p_{j}}\beta_{j,l}x_{j,t-l}$ is the DL component, and $e_{t}$ is the random error component. The ARDL is a linear time series model that considers predictors that are stationary at the level I(0), stationary at the first difference I(1), or mixed features I(0) and I(1), as well as its parameters, are estimated using the ordinary least squares (OLS) approach [70–72,92,93]. According to these features, the chosen predictor variables should be I(0) or I(1), with no strong pairwise correlation, since a strong correlation between predictors leads to spurious results of OLS estimates [70–72,92,93]. In this regard, statistical modeling guidelines were employed to choose appropriate time series variables for the ARDL model (Fig 1). This modeling technique eliminated the variables from the model that were not stationary even at the first difference or I(1). Afterward, to prevent multicollinearity, some variables (POP, FC, and EC) were excluded when their pairwise correlations with ARB were greater than | ± 0.80|.

### 3.4. ECM to predict long- and short-run relationships

The first differences of the dependent variable in equation (1) are regressed in the ECM on the first lag of the independent, dependent, and error correction term (ECT). Consequently, the ECM for this study can be stated as:


$$\Delta y_{t}=\alpha_{0}+\alpha_{1}y_{t-1}+\sum_{j=1}^{m}\alpha_{1+j}x_{j,t-1}+\sum_{i=1}^{k-1}\delta_{i}\Delta y_{t-i}+\sum_{j=1}^{m}\sum_{l=1}^{p_{j}-1}\gamma_{j,l}\Delta x_{j,t-l}+\tau ECT_{t-1}+\varepsilon_{t}\cdots \;\;\cdots \;\;\cdots \;\;\;$$ (2)

In the equation (2) $\alpha_{0}$ is the intercept; $\alpha_{1},\;\alpha_{2},\;\alpha_{3}\;\cdots \;\alpha_{m+1}$ are coefficients for the long-run relationship; $\delta_{i}s$ and $\gamma_{j,l}s$ are short-run dynamics for the dependent and independent variables, respectively, and the coefficient τ is termed as the speed of long-run adjustment or equilibrium. There are different ways to test the long-run relationship between the observed and predicted values of the dependent variable. One of them is to test the unit root of the residuals in equation (1), and the second one is to test $H_{0}:\alpha_{1}=\;\alpha_{2}=\;\alpha_{3}=\;\cdots =\alpha_{m+1}\;$ using the bound F test statistic. The negative significant value of τ specifies that the model will adjust to the long-run equilibrium.

### 3.5. Model (ARDL) adequacy checking

Verifying the fitness of the model is necessary for robust and accurate coefficient estimates. The study looked at the long- and short-run effects of the CNCFs on YBR using the ARDL and ECM models. The $R^{2}$, adjusted $R^{2}$, and F-tests were used to see how well the dependent variable was explained. Other tests included the Breusch-Godfrey test for serial correlation, the studentized Breusch-Pagan test for heteroscedasticity, the RESET test for functional misspecification, the Jarque Bera test for residual normality, and the recursive CUSUM test for model stability were also used to check model’s adequacy. Furthermore, the bound F-test and the unit root test were performed for residuals to confirm the presence of a long-term cointegration relationship between the dependent variable’s anticipated and observed values.

### 3.6. Granger causality analysis and PCA

In this study, while fitting the ARDL model of YBR on CNCFs, due to multicollinearity issues, some non-climatic factors were removed to enhance its credibility and accuracy in forecasting the long- and short-run relationship between YBR and CNCFs. However, eliminating data or variables may result in the loss of important information [94–96], and the eliminated NCFs may have a causal relationship with YBR. Therefore, to forecast these sorts of relationships, we used Granger causality [80] and principal component analysis (PCA) [77,97]. The Granger causality analysis predicts the causal effects of one variable on another and vice versa, whether the variables are cointegrated or not [72,80–82]. On the other hand, when there is a strong pairwise correlation between CNCFs, the impact assessment of CNCFs on YBR using the OLS-based ARDL model suffers from an accuracy problem [71,73]. In this regard, PCA can be an alternative approach for assessing the relationship between YBR and CNCFs when time series variables were stationary at I(0) or I(1) [71,77]. In this case, regressing YBR on the important PC scores, the CNCFs that have a substantial impact on YBR can be retrieved from the contribution of the CNCFs to the respective PC.

## 4. Results

### 4.1. Descriptive statistics, stationarity checking, and correlation analysis

**Fig 2:**
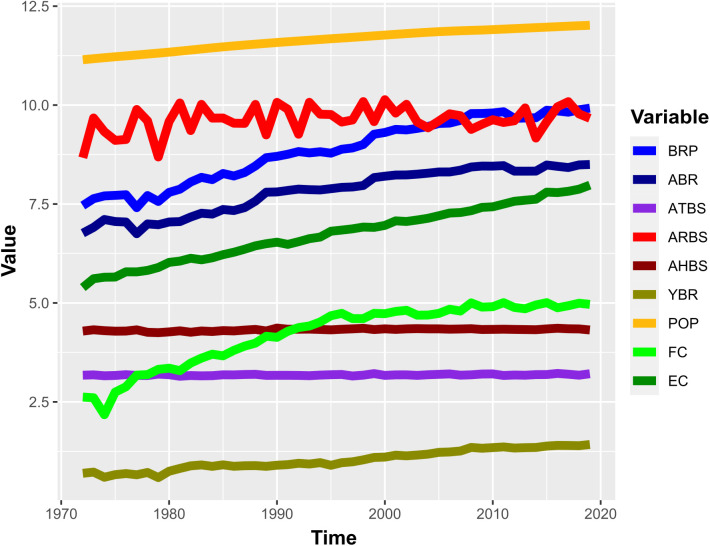
Time series plot of the variables BRP, ABR, YBR, ATBS, ARBS, AHBS, POP, FC, and EC.

This study analyzed descriptive statistics to obtain summary information about the CNCFs, while stationarity checking and correlation analysis were conducted to choose CNCFs in the subsequent analysis. Accordingly, Table 3 reports the results of descriptive statistics like mean, SD, skewness, and so on. The descriptive statistics mean±SD of the CNCFs: BRP, ABR, YBR, ATBS, ARBS, AHBS, POP, FC, and EC were 8.85 ± 0.82, 7.82 ± 0.57, 1.03 ± 0.25, 3.18 ± 0.02, 9.63 ± 0.34, 4.32 ± 0.03, 11.65 ± 0.26, 4.21 ± 0.80, and 6.75 ± 0.71. Besides these summary statistics, Table 3 displays the maximum, minimum, median, Q_1_, Q_2_, and skewness. Fig 2 provides a pictorial view of the CNCFs to grasp the structure of the time series variables.

**Table 3:** Summary statistics of the considered time series variables.

Statistic	BRP	ABR	YBR	ATBS	ARBS	AHBS	POP	FC	EC
Minimum	7.41	6.75	0.59	3.15	8.67	4.25	11.15	2.18	5.39
Q_1_	8.16	7.26	0.87	3.17	9.53	4.30	11.44	3.65	6.14
Median	8.86	7.90	0.96	3.18	9.62	4.33	11.69	4.61	6.83
Mean	8.85	7.82	1.03	3.18	9.63	4.32	11.65	4.21	6.75
Q_3_	9.63	8.33	1.28	3.19	9.89	4.34	11.88	4.84	7.30
Maximum	9.92	8.50	1.42	3.22	10.14	4.37	12.02	5.01	7.98
SD	0.82	0.57	0.25	0.02	0.34	0.03	0.26	0.80	0.71
Skewness	−0.27	−0.42	0.04	0.35	−0.81	−0.80	−0.34	−0.67	−0.06

This study also checked stationarity using the ADF and PP tests, and the Zivot-Andrews and Minimum Lagrange Multiplier unit root tests were used to identify single and multiple SB in the time series data. The results of these tests are shown in Tables 4–6. At the 5% level of significance, The ADF and PP test results showed that YBR, ARBS, AHBS, and EC were I(1) in the ADF test and I(0) in the PP test, ABR and FC were I(1) in both the ADF and the PP test, and POP was stationary at I(1) in the ADF test (Table 4). The Zivot-Andrews Unit Root Test (Table 5) showed that the YBR, ABR, and POP remained stationary at I(1) even with SB in 1981, 1989, and 2004, respectively, while the ATBS, ARBS, AHBS, FC, and EC remained stationary with SB in 1980, 2002, 1986, 1994, and 2001, respectively. Additionally, even with two SB points, all the CNCFs were stationary at the 1% significance level, according to the findings of the Minimum Lagrange Multiplier Unit Root Test (Table 6).

**Table 4:** Stationarity testing of the time series variables using ADF and PP tests.

Variable	P-Value (ADF Test)		P-Value (PP Test)	
	I(0)	I(1)	I(0)	I(1)
YBR	0.250	0.010	0.026	0.010
ABR	0.944	0.023	0.677	0.010
ATBS	0.012	0.010	0.010	0.010
ARBS	0.299	0.010	0.010	0.010
AHBS	0.790	0.033	0.010	0.010
POP	0.959	0.010	0.990	0.466
FC	0.717	0.010	0.955	0.010
EC	0.054	0.010	0.010	0.010

**Table 5:** Stationarity testing of the time series variables in the presence of a single SB using the Zivot-Andrews Unit Root Test.

Variable	I(0)		I(1)	
	t-statistic	SB	t-statistic	SB
**YBR**	−4.19	1985	−11.41***	1981
**ABR**	−3.94	1987	−6.34***	1989
**ATBS**	−6.77***	1980	−8.88***	1975
**ARBS**	−9.32***	2002	−11.84***	2014
**AHBS**	−6.24***	1986	−12.24***	1978
**POP**	−3.50	1981	−5.21**	2004
**FC**	−5.64***	1994	−13.35***	1975
**EC**	−6.72***	2001	−9.44***	2013

Note: *** and ** indicate significance at 1% and 5% levels, respectively.

**Table 6:** Stationarity testing of the time series variables in the presence of multiple SB using the Minimum Lagrange Multiplier Unit Root Test. In tables SB(1) and SB(2) represent the year of the first and second SB, respectively.

Variable	I(0)			I(1)		
	t-statistic	SB(1)	SB(2)	t-statistic	SB(1)	SB(2)
**YBR**	−5.27***	1981	1997	−7.65***	1977	1994
**ABR**	−6.78***	1993	1997	−8.06***	1987	1990
**ATBS**	−7.06***	1982	1987	−10.08***	1994	1997
**ARBS**	−9.12***	1979	2007	−10.96***	1989	1992
**AHBS**	−8.24***	1978	1986	−11.92***	1978	1997
**POP**	−7.00***	1986	2002	−5.29***	1988	2004
**FC**	−7.72***	1988	1992	−9.47***	1996	2003
**EC**	−6.34***	1991	1997	−8.15***	1977	2004

Note: *** indicates significance at 1% level.

The results of the correlation analysis were reported in Table 7, which indicated that all the variables are positively correlated with BRP and YBR. From the result, it can be concluded that there was no strong pairwise correlation among the predictors ABR, ATBS, ARBS, and AHBS. However, the pairwise correlation of POP, FC, and EC with ABR was very high (±0.80) which may cause multicollinearity problems in further analysis.

**Table 7:** Pairwise correlation of the Time series variables BRP, ABR, YBR, ATBS, ARBS, AHBS, POP, FC, and EC.

	BRP	ABR	YBR	ATBS	ARBS	AHBS	POP	FC	EC
**BRP**	1								
**ABR**	0.994	1							
**YBR**	0.970	0.939	1						
**ATBS**	0.417	0.405	0.425	1					
**ARBS**	0.312	0.312	0.299	−0.084	1				
**AHBS**	0.738	0.761	0.655	0.207	0.517	1			
**POP**	0.989	0.981	0.964	0.421	0.342	0.720	1		
**FC**	0.944	0.951	0.889	0.382	0.373	0.718	0.966	1	
**EC**	0.978	0.963	0.971	0.446	0.326	0.685	0.991	0.934	1

### 4.2. Fitting ARDL model for YBR

In this section, the predictors were selected to evaluate the impact of CNCFs on YBR using the ARDL model. Despite the stationary nature (I(0) or I(1)) of all the variables, this study excluded POP, FC, and EC from the ARDL model because of their strong pairwise correlation with ABR. This study, therefore, incorporated the most important predictors, ABR, ATBS, ARBS, and AHBS, into the ARDL model. Next, this study fitted the ARDL model of YBR on the predictors ABR, ATBS, ARBS, and AHBS using the Akaike information criterion (AIC). Consequently, we found the optimum ARDL (4, 2, 4, 0, 2) model for the lowest AIC value (−164.6652). In this model, the optimum lags for the YBR, ABR, ATBS, ARBS, and AHBS were 4, 2, 4, 0, and 2, respectively.

### 4.3. Estimation of long- and short-run coefficients and cointegration relationship

**Fig 3:**
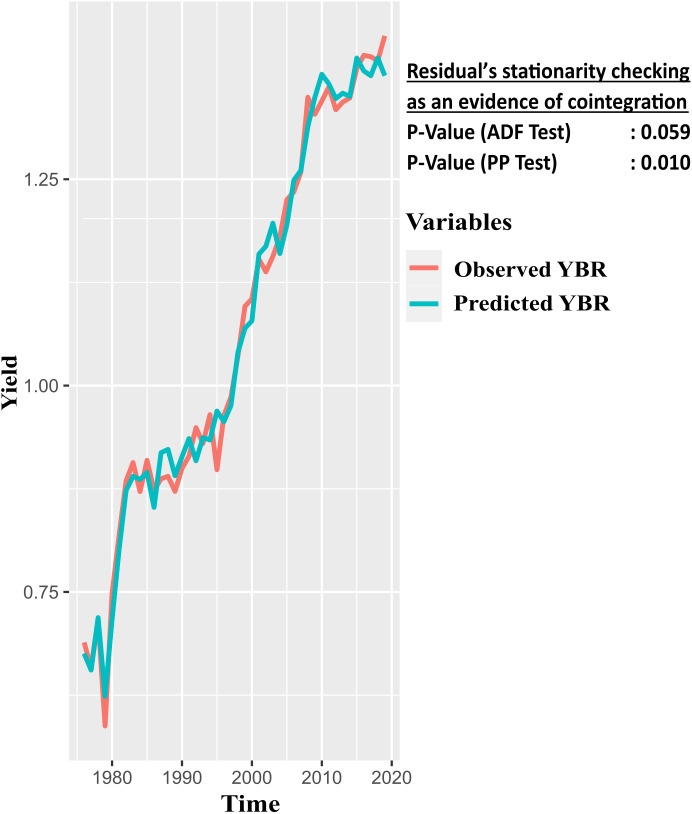
Cointegration plot of observed versus predicted YBR.

To investigate the long-run relationships between the observed and predicted values of YBR, we utilized the ARDL bound test and the unit root test for error analysis. This study presented the test results in Table 8 and Fig 3. According to these results, the ARDL bound testing approach demonstrated that the computed F-statistics of 25.28 exceeded the upper bound critical value at the 1%, 5%, and 10% significance levels (Table 7). The ADF and PP tests for the unit root and the stationarity residuals of the ARDL model also showed cointegration between the predicted and observed YBR (Fig 3). Therefore, this result confirms that there is a strong long-term cointegration relationship among YBR, ABR, ATBS, ARBS, and AHBS.

**Table 8:** ARDL bound test results for cointegration.

F-Statistic Value	Level of significance	I(0)	I(1)	P-Value
25.28	10%	2.2191	3.0968	0.000
	5%	2.5680	3.4937	0.000
	1%	3.3354	4.3614	0.000

Table 9 reports a significant positive impact of ABR and ATBS, a significant negative impact of AHBS, and an insignificant negative impact of ARBS on YBR in the long-run. Interpretively, a 1% increase in ABR and ATBS would increase the YBR by 0.602% and 5.985%, respectively. On the other hand, a 1% increase in AHBS would result in a 5.4% decrease in the yield.

**Table 9:** Results of long-run relationships based on ECM following the ARDL (4, 2, 4, 0, 2) model, where YBR is the dependent variable.

Variables	Coefficients	SE	t-Statistic	P-Value
(Intercept)	0.7041	11.2295	0.0627	0.9500
ABR	0.6022	0.0792	7.6020	0.0000
ATBS	5.9853	2.7770	2.1553	0.0402
ARBS	−0.0068	0.0732	−0.0932	0.9260
AHBS	−5.4003	1.3665	−3.9517	0.0005

On the other hand, Table 10 reports the short-run relationships between the YBR and the climatic factors. The results showed that, with every 1% increase in ABR, YBR increased by 0.298%. However, in the prior period, for the 1% increase in the ABR, there was a 0.334% drop in the YBR compared to the current period. ATBS had a significant negative impact on YBR during its initial lag, suggesting a substantial drop of 1.446% in YBR. However, it has a significant positive short-run effect on YBR alongside a coefficient of 0.818, reflecting an increase in YBR of 0.818% for every 1% increase in ATBS. Furthermore, YBR declines were explained by the second and third lags in ATBS, with the third lag demonstrating statistical significance. On the contrary, during the Boro rice-producing season, AHBS positively impacted YBR in the short run, with a coefficient of 0.594 indicating a 0.594% increase in YBR for every 1% increase in AHBS. Its one-period lag indicated that a 1% increase in AHBS in the preceding period is linked to a significant increase of 1.176% in the YBR in the current period. The first and second lags of YBR have a considerable positive impact on the present YBR. A 1% increase in the first and second lags of YBR results in a 0.26% and 0.244% increase in the current year YBR at the 10% and 5% significance level, respectively. Finally, the rate of adjustment to the long-run equilibrium after a shock in the previous period is indicated by the negative coefficient of the lagged ECT. A disturbance in the prior period significantly readjusts to equilibrium at a long-term rate of 0.3641%, according to the coefficient of ECT.

**Table 10:** Results of short-run dynamics based on ECM following ARDL (4, 2, 4, 0, 2) model where YBR is the dependent variable.

Variables	Coefficients	SE	t-Statistic	P-Value
(Intercept)	0.2564	0.0509	5.0278	0.0001
d(L(YBR, 1))	0.2605	0.1496	1.7412	0.0915
d(L(YBR, 2))	0.2439	0.1127	2.1626	0.0383
d(L(YBR, 3))	−0.0171	0.1058	−0.1624	0.8720
d(ABR)	0.2977	0.0611	4.8674	0.0000
d(L(ABR, 1))	−0.3338	0.0722	−4.6185	0.0001
d(ATBS)	0.8177	0.3401	2.4038	0.0223
d(L(ATBS, 1))	−1.4460	0.4008	−3.6077	0.0010
d(L(ATBS, 2))	−0.6215	0.3521	−1.7649	0.0874
d(L(ATBS, 3))	−0.6602	0.3143	−2.1001	0.0439
d(AHBS)	0.5940	0.2849	2.0845	0.0454
d(L(AHBS, 1))	1.1760	0.2859	4.1130	0.0002
ect	−0.3641	0.0765	−4.7591	0.0004

### 4.4. Results of model adequacy checking

**Fig 4:**
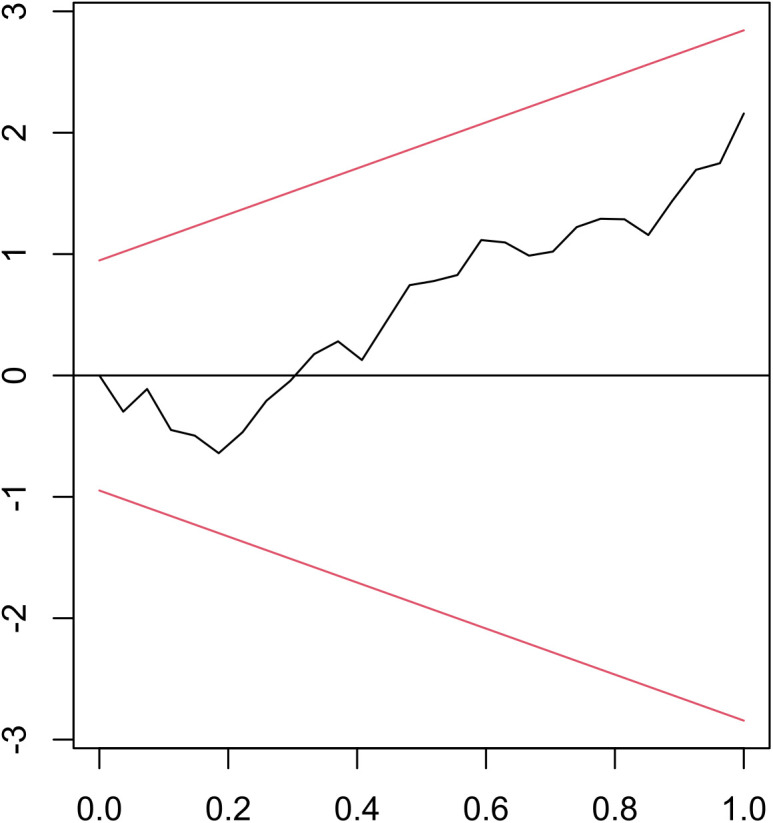
CUSUM plot for model stability checking.

The tests of model adequacy present an extensive assessment of its assumptions and goodness of fit (Table 11). The remarkably high R^2^ value of 0.989 and the adjusted R^2^ of 0.982 point to a well-fitted ARDL (4, 2, 4, 0, 2) model, with the data suggesting that the variables included explain approximately 98% of the variation in YBR. Additionally, the F-test supports the model’s overall significance with a highly significant result of 151.70 at the 1% significance level. The Breusch-Godfrey test result for serial correlation showed an insignificant value of 2.959 (P-value = 0.5646), indicating that serial correlation is absent in the residuals. Heteroscedasticity was absent in the residuals, as shown by the insignificant result of 9.914 (P-value = 0.871) from the Studentized Breusch-Pagan test. A non-significant result of 0.068 (P-value = 0.7963) from the RESET test for functional misspecification indicated that the model is not mis specified. The Jarque-Bera test for residual normality produces a non-significant value of 0.933 (P-value = 0.627), indicating that the residuals are normally distributed. Similarly, a non-significant result of 0.719 (P-value = 0.2237) is obtained using the Recursive CUSUM test, indicating that the coefficients have been stable throughout time (Fig 4). Overall, the results of all the diagnostic tests support the validity and suitability of the fitted optimum ARDL (4, 2, 4, 0, 2) model for examining the dynamics of YBR in response to YBR, ABR, ATBS, ARBS, and AHBS.

**Table 11:** Results of the model diagnostic test statistic.

Diagnostic test statistic	Values	P-Value
R^2^	0.989	
Adjusted R^2^	0.982	
F-Test	151.70	0.0000
Breusch-Godfrey test for serial correlation	2.959	0.5646
Studentized Breusch-Pagan test for heteroscedasticity	9.914	0.871
RESET test for functional misspecification	0.068	0.7963
Jarque Bera Test for residuals normality	0.933	0.627
Recursive CUSUM test	0.719	0.2237

### 4.5. Results of Granger causality analysis and PCA

**Fig 5:**
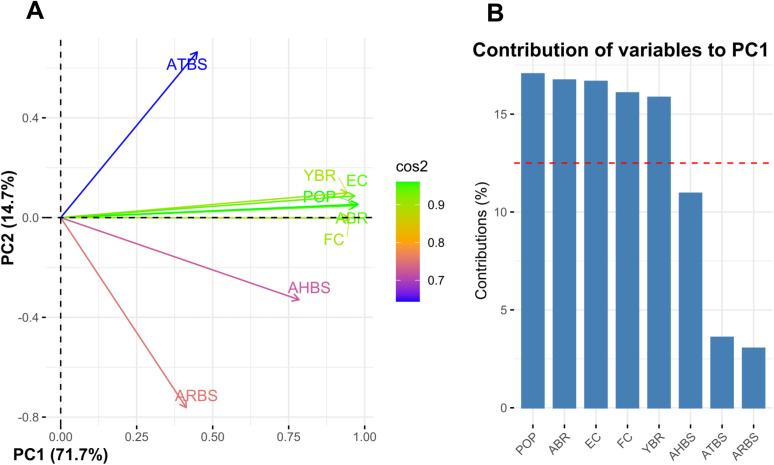
The PCA of CNCFs and YBR. Figure A represents the biplot of the CNCFs for PC1 and PC2, and Figure B represents the contribution of the CNCFs to PC1.

This section used Granger causality to investigate the causal association between YBR and CNCFs (Table 12). Table 12 revealed a significant unidirectional causal relationship between ABR and ATBS to YBR and YBR to AHBS. The non-climatic factors POP, FC, and EC also showed a significant unidirectional causal association with YBR. This evidence indicated that all the CNCFs except ARBS significantly impact YBR in Bangladesh. On the other hand, PCA was conducted to identify the impact of non-climatic factors on YBR, as they were eliminated from the ARDL model due to high pairwise correlation. PCA is a statistical method that extracts the most relevant features or variables by transforming the initial stationary data into a lower-dimensional space and grouping the highly correlated variables [74–79]. In this study, together, PC1 (71.7%) and PC2 (14.7%) accounted for 86.4% of the variance in the original data (Fig 5A). According to the biplot (Fig 5A), YBR and the non-climatic components YBR, ABR, POP, EC, and FC clustered together and displayed the greater distance from the origin on PC1’s positive quadrant. At the same time, ABR, POP, EC, FC, and YBR all exceeded the expected average contribution threshold (red dashed line) to PC1 in Fig 5B. This suggests that the non-climatic factors have a positive impact on YBR because they contribute significantly to the variation of PC1 (71.7%). Therefore, from the PCA analysis, it could be concluded that non-climatic factors ABR, POP, EF, and EC positively impacted YBR. Simultaneously, the regression analysis results in Table 13 show that the scores of PC1 and PC2 efficiently explained the variation (92%) in YBR with residual SE 0.074.

**Table 12:** Results of the Granger causality analysis of YBR on the CNCFs.

Null Hypothesis	F-Statistic	P-Value	Decision
ABR does not Granger cause YBR	2.6911	0.100	H_0_ Rejected
YBR does not Granger cause ABR	1.1231	0.295	H_0_ Accepted
ATBS does not Granger cause YBR	3e-04	0.987	H_0_ Accepted
YBR does not Granger cause ATBS	7.5639	0.009	H_0_ Rejected
ARBS does not Granger cause YBR	0.902	0.347	H_0_ Accepted
YBR does not Granger cause ARBS	2.6035	0.114	H_0_ Accepted
AHBS does not Granger cause YBR	0.0046	0.946	H_0_ Accepted
YBR does not Granger cause AHBS	4.8146	0.034	H_0_ Rejected
POP does not Granger cause YBR	9.370	0.003	H_0_ Rejected
YBR does not Granger cause POP	1.5702	0.213	H_0_ Accepted
FC does not Granger cause YBR	4.001	0.090	H_0_ Rejected
YBR does not Granger cause FC	0.022	0.883	H_0_ Accepted
EC does not Granger cause YBR	10.558	0.002	H_0_ Rejected
YBR does not Granger cause EC	1.7522	0.1924	H_0_ Accepted

**Table 13:** Regression analysis of YBR on PC1 and PC2 scores.

Variables	Coefficients	SE	t-Statistic	P-Value
Intercept	1.031	0.011	96.910	2e^-16^
PC1 Scores	10.101	0.004	22.500	2e^-16^
PC2 Scores	20.023	0.010	2.370	0.0221
R^2^	0.919			
Adjusted R^2^	0.916			
Residual SE	0.074			
F-Statistic	256			2.2e^-16^

## 5. Discussion

Though rice is important in the context of economy and health, there is a great reduction in its yield due to the impact of CC, which threatens food security everywhere, including in Bangladesh [1,5,21]. At the same time, some of the non-climatic factors ABR, POP, FC, and EC enhance production [28–31,33]. Therefore, policies should be implemented to keep climatic conditions at a congenial state during Boro rice production, and the judicious use of non-climatic factors is required for sustainable rice production. Accordingly, this study attempted to assess the impact of CNCFs on YBR employing time series modeling approaches for effective policy implications. Subsequently, this study considered the predicted variable YBR with multiple predictors (BRP, ABR, ATBS, ARBS, AHBS, POP, FC, and EC), all of which were either I(0) or I(1) to fit the ARDL model. However, since the ARDL model estimates the coefficients using the OLS method, a high pairwise correlation in the predictors results in multicollinearity issues [70–72,92,93]. Subsequently, due to their high correlation with ABR, the fitted ARDL model eliminated a few non-climatic factors, such as POP, FC, and EC. However, removing variables or data may result in the loss of crucial information [94–96], and the omitted non-climatic factors may have a causal relationship with YBR. Therefore, the Granger causality test was used in this study to investigate the causal relationship between YBR and CNCFs, which is a widely used method for analyzing causal relationships between time series variables, regardless of their cointegration [72,80–82]. Furthermore, PCA is an alternative multivariate method for evaluating the relationship between YBR and CNCFs, even when multicollinearity problems occur in the predictors. This method can also be used with time series data when time series variables are stationary at I(0) or I(1), [74–79]. Finally, the time series modeling includes the ARDL model, the Granger causality test, and PCA to predict the impact of CNCFs on YBR.

The ARDL-based ECM identified a long- and short-run relationship between YBR and ABR, ATBS, ARBS, and AHBS. ABR eventually had a significant positive impact on YBR in the long-run, and its first lag negatively impacted YBR. These results are also supported by the literature, which shows that the growing trend in the area of planting rice or other cereals has increased yields in several countries, including Bangladesh [52,53,65,67,98–100]. Additionally, the adoption of high-yielding Boro rice varieties, technological advancement, expansion of extension work and research, and government subsidies for fertilizer and irrigation systems increased the rice acreage and yield [59,65,101–103]. In this issue, the governments and agricultural bodies should implement policies to support agricultural expansion and rice yield enhancement by converting barren land into arable land, incentivizing efficient irrigation, protecting farmers from natural disasters, providing scientific knowledge for mechanized farming, and using AI, sensors, and drones for crop health monitoring. This study also found that ATBS had a distinctly positive long- and short-run impact on YBR, which is believed to be the primary indicator of the CC [104,105]. Conversely, YBR suffered from the first and third lags of ATBS. These results are also supported by literature, and usual temperature prevails over the Boro rice growing season in Bangladesh. Accordingly, Bangladesh’s rice varieties thrive in 20°C-30°C temperatures, with ideal day and night temperatures of 20°C-36°C and 20°C-23°C for optimal growth [106,107]. Nevertheless, the average minimum and maximum temperatures throughout the Boro season (November to May) range from 18.50 ° C to 29.50^o^ C [60,61,108]. As a result, the temperature during the Boro season in Bangladesh prevails at a congenial state for rice production, which positively impacts the YBR. Additionally, the current development of molecular science and bioinformatics has developed different heat-tolerant and high-yielding rice varieties [7,109–113], which sustains an increase in YBR. In addition, some investigations confirm our results [114–116], while others show the opposite [67,117]. Although the current temperature is friendly for rice cultivation, its tendency to rise will endanger both rice production and food security in the future. Thus, to continue a desirable global temperature, national and international policies should be initiated that limit greenhouse gas emissions, improve carbon sequestration, raise public awareness, encourage personal lifestyle changes, etc. In addition, other steps such as the creation of heat-tolerant crops, sustainable land management, effective water use, early warning systems, and farmer subsidies should be implemented to produce rice sustainably. In contrast, this study did not observe any significant impact of ARBS on YBR for either short- or long-term relationships. These results were also consistent with those of Granger Causality analysis. This is because, in Bangladesh, BRP depends on irrigation since rainfall during this season is only approximately 6% of the yearly total [118,119]. Nonetheless, several studies [42,120,121] observed that rainfall negatively influences rice production and other cereals, while gradually rising rainfall during the flowering and harvesting stages causes serious yield loss [8,120,122]. Although AHBS has a short-run beneficial impact on YBR, it has a significant long-run adverse effect. Other scientific investigations have validated these findings. In the short-run, higher humidity can benefit rice production during the winter by promoting greater growth, improved nutrient uptake, and photosynthesis [123,124]. However, in the long run, high humidity combined with temperature and precipitation causes an increase in diseases such as bacterial leaf blight and rice blast, as well as insect stress and negative impacts on the grain-filling process that ultimately result in decreased production [99,123–126].

The Granger causality test identified a causal relationship between the non-climatic factors ABR, POP, FC, and EC to YBR, as well as from YBR to climatic factors ATBS and AHBS. Therefore, all the CNCFs except ARBS have a causal effect on YBR. On the other side, the non-climatic factors YBR, ABR, POP, EC, and FC substantially contributed to PC1’s (71.7%) variation, clustered together, and placed on PC1’s positive quadrant (Fig 5). Therefore, the non-climatic factors ABR, POP, EC, and FC positively impacted YBR. The correlation analysis also supported that POP, FC, and EC, the eliminated non-climatic factors from the ARDL model, were positively associated with YBR. The study’s findings on the relationship between the non-climatic factors and YBR aligned with Boserup’s Theory of Agricultural Intensification [28], which states that population growth pushes farmers to adopt more efficient and productive agricultural practices like fertilizer use and the adoption of modern technologies for more production. Nevertheless, regarding FC and EC, though our results align with other studies [67,127], others found a detrimental impact on maize production [42].

Finally, aligning the results of the ARDL model, Granger causality, and PCA, this study found that the ABR, ATBS, POP, EC, and FC substantially positively impacted the YBR in Bangladesh, while AHBS impacted negatively. Other findings also support these conclusions [13,44,51–53,56,128]. These discussions clearly show that the fitted ARDL model, ECM, Granger causality test, and PCA yielded results consistent with the literature in the context of Bangladesh and other nations. Consequently, these findings have provided significant insights for policymakers and stakeholders to formulate effective strategies for sustainable rice production during the Boro season.

### 5.1. Limitations of the study and scope of future research

This study provides valuable insights on the impact of CNCFs on YBR, and policies taken based on these insights may pave the way for sustainable rice production, specifically YBR and food security. However, this study also has some limitations. Usually, there are three rice-growing seasons in Bangladesh: Aus, Aman, and Boro, with distinct climatic conditions. Among these three rice types, Boro rice is the most important seasonal rice in terms of production yield and acres, which is the concern of this study. Therefore, the impact of CNCFs on Aus and Amon remains unexplored. The climatic and non-climatic factors often interplay for better rice yield. For example, rice and other crops respond to changes in temperature, water availability, and CO_2_ concentrations in complex ways. Rising CO_2_ can stimulate photosynthesis, potentially increasing yields, but water, nutrient availability, and temperature stress in response to rising CO_2_ modulate this effect, making it difficult to isolate one factor’s impact. The non-climatic factors, like the adaptation rate of farmers to changing conditions (e.g., shifting planting times, adopting new technologies, irrigation), changing market demand, input costs, and rate of pesticide use, are also important factors that influence the YBR. The lack of suitable data on these factors prevented their inclusion in this study. Reliable and efficient prediction often depends on the consistency of high-quality, historical data on crop yields and CNCFs. Consequently, the prediction of this would be more robust if more consistent historical data could be ensured. Methodological limitations, on the other hand, necessitated using multiple statistical methods to evaluate the impact of CNCFs, making the comparison of results somewhat challenging. Given these limitations, future research could incorporate a wide range of more consistent, suitable CNCFs for robust prediction of CNCFs’ impact on YBR. Additionally, the researcher should focus on developing more flexible and reliable statistical algorithms/methods for users to predict the impact of multiple climatic and non-climatic factors on crop production, including the seasonal rice production comprehensively.

## 6. Conclusions and policy implications

This study explored the impacts of CNCFs on YBR in Bangladesh using time-series modeling approaches based on data from 1972 to 2019. The modeling approaches employed ADF and PP tests to verify the unit root and the suitability of CNCFs for subsequent analyses. The Zivot-Andrews and Minimum Lagrange Multiplier Unit Root Tests were utilized to confirm single and multiple SB within the CNCFs. Then, correlation analysis was used to ascertain the presence of a strong pairwise correlation between the predictors. The ARDL-based ECM was then used to predict the long- and short-run impact of CNCFs on YBR. The causal relationships between the YBR and CNCFs were predicted using the Granger causality test. Due to methodological limitations, the ARDL model is insufficient to assess the impact of all the CNCFs, so PCA was used to predict the effect of the rest of the CNCFs on YBR. Finally, aligning the results of all these analyses, the impact of all the CNCFs on YBR was assessed collectively. The ADF and PP tests found the CNCFs stationary at I(0) or I(1). Consequently, the CNCFs were suitable candidates for the ARDL model, Granger causality, and PCA. However, the non-climatic factors POP, FC, and EC were removed from the ARDL model due to their strong pairwise correlation with ABR. Therefore, this study fitted an optimized ARDL (4, 2, 4, 0, 2) model with YBR, ABR, ATBS, ARBS, and AHBS when AIC was at its minimum (−164.67), which explained 98.9% of the overall variation in YBR. The fitted ARDL-based ECM projected that AHBS had a long-term detrimental impact on YBR, while ABR and ATBS had a considerable positive impact. These factors also positively impacted YBR in the short run. However, ARBS showed an insignificant effect on YBR in the long- and short-run. The Granger causality study revealed a strong unidirectional causal association between CNCFs and YBR, except for ARBS. In addition, the PCA results support the favorable effects of the non-climatic factors ABR, POP, EC, and FC on YBR. Aligning these results, this study found that ABR, ATBS, POP, FC, and EC positively and AHBS negatively impacted YBR in Bangladesh.

The insights of this study suggested the implementation of the following policies.

1. The ever-increasing population puts extreme pressure on limited arable land to produce more crops. Consequently, the productivity of land decreases due to the extensive use of synthetic fertilizers. Therefore, encouraging people to use family planning and other population management strategies should be implemented to keep the population in an affordable state.
2. Modern technology-based rice and BR farming practices should be implemented for sustainable production and food security. The modern farming practice includes precision farming, balanced fertilizer use, sustainable land and water management techniques, and soil health preservation.
3. Policies should be implemented at the national and international levels to reduce greenhouse gas emissions to maintain a balanced ATBS and AHBS, as extreme levels can harm rice production. Additionally, localized weather monitoring and farmers’ training are required for sustainable rice and BR production.
4. Fertilizer intake positively impacts YBR. However, the abuse of synthetic fertilizers will eventually reduce the soil’s fertility. Thus, the government ought to encourage farmers to use organic fertilizers and start training them in precision farming and the judicious use of chemical fertilizers.
5. Although energy consumption positively affects YBR, its excessive consumption results in higher emissions of some air pollutants and greenhouse gases. Therefore, the government should support and subsidize energy-efficient and environmentally friendly technologies and the implementation of eco-taxation measures to promote the utilization of renewable energy sources.
6. Finally, together with the implementation of the above policies, policymakers and researchers should focus on developing climate-resilient and short-duration rice cultivars for sustainable rice production and food security.

## Supporting information

S1 Appendix The R packages are used for data analysis. (DOCX)

S1 File Supporting information. (ZIP)
