# Supplementary material for: Impact of climate change on Boro rice production in Bangladesh: Evidence from time series modeling
Source: PLoS One. 2025 Jul 23;20(7):e0328699. doi: 10.1371/journal.pone.0328699 (PMC12286385; doi:10.1371/journal.pone.0328699)
Supplement: S1 Appendix — (DOCX) [file pone.0328699.s001.docx]

***Appendix***

**Impact of climate change on Boro rice production in Bangladesh: evidence from time series modeling**

**Rafee Shahrier^1,3^, Mohammad Nazmol Hasan^2*^ Sadia Yesmin Ankita^1,4^, Ismat Tasnim^1,5^, and Kazi Tamim Rahman^6^**

^1^Faculty of Agricultural Economics and Rural Development, Gazipur Agricultural University, Gazipur-1706, Bangladesh.

^2^Department of Agricultural and Applied Statistics, Gazipur Agricultural University, Gazipur-1706, Bangladesh.

^3^Department of Agricultural and Applied Statistics, Bangladesh Agricultural University, Mymensingh-2202, Bangladesh.

^4^Department of Agricultural Statistics, Sher-e- Bangla Agricultural University, Dhaka-1207, Bangladesh.

^5^Department of Agribusiness and Marketing, Bangladesh Agricultural University, Mymensingh-2202, Bangladesh.

^6^Department of Agricultural Economics, Gazipur Agricultural University, Gazipur-1706, Bangladesh.

**^*^Corresponding author:** Mohammad Nazmol Hasan, Email: [nazmol.stat.bioin@gau.edu.bd](mailto:nazmol.stat.bioin@bsmrau.edu.bd)

**Appendix**

We utilized specific packages in the R programming software to analyze the various statistical methods employed in the article. Below is a description of the statistical techniques and sources of R packages.

1. The pairwise correlation between the variables was conducted using the R package “GGally”[1].
2. The Augmented Dickey Fuller (ADF) and Phillips-Perron (PP) tests were conducted using the R package “tseries” [2].
3. The Zivot-Andrews Unit Root Test was conducted using the R package “urca” [3].
4. The Minimum Lagrange Multiplier Unit Root Test was conducted using a R function from a GitHub repository “LeeStrazicichUnitRoot” from the source “<https://github.com/hannes101/LeeStrazicichUnitRoot/blob/master/LeeStrazicichUnitRootTest.R>”.
5. The ARDL model was fitted using the R package “ARDL” [4].
6. The Granger causality test was conducted using the R package “lmtest” [5].
7. The PCA was conducted using the R Package “stats”.
8. PCA biplot and variable contribution to PCs plot require R packages "FactoMineR", "factoextra", ggplot2, and “gridExtra”.

**References**

1. Schloerke B, Cook D, Larmarange J, Briatte F, Marbach M, Thoen E, et al. Ggally: Extension to ggplot2. In: R package version 0.5.0. 2020.

2. Trapletti A, Hornik K. tseries: Time Series Analysis and Computational Finance. R Packag version 010-42. 2017.

3. Zivot E, Andrews DWK. Further Evidence on the Great Crash, the Oil-Price Shock, and the Unit-Root Hypothesis. J Bus Econ Stat. 1992;10: 251. doi:10.2307/1391541

4. Natsiopoulos K, Tzeremes NG. ARDL: An R package for the analysis of level relationships. J Open Source Softw. 2022;7. doi:10.21105/joss.03496

5. Hothorn T, Zeileis A, Farebrother RW, Cummins C, Millo G, Mitchell D. R Package ‘ lmtest ’: Testing Linear Regression Models. R News. 2018.
